# Lon-dependent proteolysis in oxidative stress responses

**DOI:** 10.1128/jb.00005-25

**Published:** 2025-06-06

**Authors:** Kubra Yigit, Peter Chien

**Affiliations:** 1 Department of Biochemistry and Molecular Biology, Molecular and Cellular Biology Program, University of Massachusetts, Amherst, Massachusetts, USA; The Ohio State University, Columbus, Ohio, USA

**Keywords:** AAA+ protease, Lon, oxidative stress, carbonylation, quality control, iron homeostasis, redox, mitochondria, bacteria

## Abstract

Accumulation of reactive oxygen species (ROS) induces oxidative stress, leading to substantial damage to cellular macromolecules, necessitating efficient protein quality control mechanisms. The Lon protease, a highly conserved ATP-dependent protease, is thought to play a central role in mitigating oxidative stress by targeting damaged and misfolded proteins for degradation. This review examines the role of Lon in oxidative stress responses, including its role in degrading oxidized proteins, regulating antioxidant pathways, and modulating heme and Fe-S cluster homeostasis. We highlight cases of substrate recognition through structural changes and describe situations where Lon activity is further regulated by redox conditions. By synthesizing studies across a range of organisms, we find that despite the clear importance of Lon for oxidative stress tolerance, universal rules for Lon degradation of damaged proteins during this response remain unclear.

## INTRODUCTION

Oxidative stress arises from an excess of reactive oxygen species (ROS), reactive nitrogen species (RNS), and free radicals, overwhelming the cell’s antioxidant defenses (1). ROS, such as superoxide (O_2_^-^) and hydrogen peroxide (H_2_O_2_), are natural byproducts of metabolism and essential for various cellular functions, but their excess can damage proteins, lipids, and DNA (2). Cells have developed antioxidant systems, including enzymes and non-enzymatic antioxidants, to mitigate this damage (3, 4). However, when ROS levels overwhelm these defense mechanisms, cumulative cellular damage can lead to dysfunction and disease. Decades of research suggest that the Lon protease plays a critical role in addressing oxidative damage in both eukaryotic and prokaryotic cells (5–8). The presence of reactive molecules, including ROS, in the cellular environment can interfere with the folding of newly synthesized proteins or compromise the structural integrity of fully folded proteins. Over time, this can cause various structural modifications that may lead to misfolding (9, 10). Although misfolded proteins are degraded by the proteasome in eukaryotes, prokaryotes rely on AAA+ (ATPases associated with diverse cellular activities) proteases such as Lon for this task (11). Given the well-known sensitivity of protein structure to oxidative damage, the role of Lon protease in oxidative stress tolerance has always been attributed to its ability to clear oxidized proteins. This review explores whether this ability can be observed in all systems during oxidative stress. We begin by describing the molecular damage caused by oxidative stress and what is known about the role of Lon in managing this stress response. We highlight known examples of Lon’s degradation of oxidized proteins and its proteolytic control of cellular pathways across kingdoms of life. We end with speculations on the conserved connection between oxidative stress-dependent protein quality control and proteases.

## OXIDATIVE DAMAGE OF MACROMOLECULES

**Fig 1:**
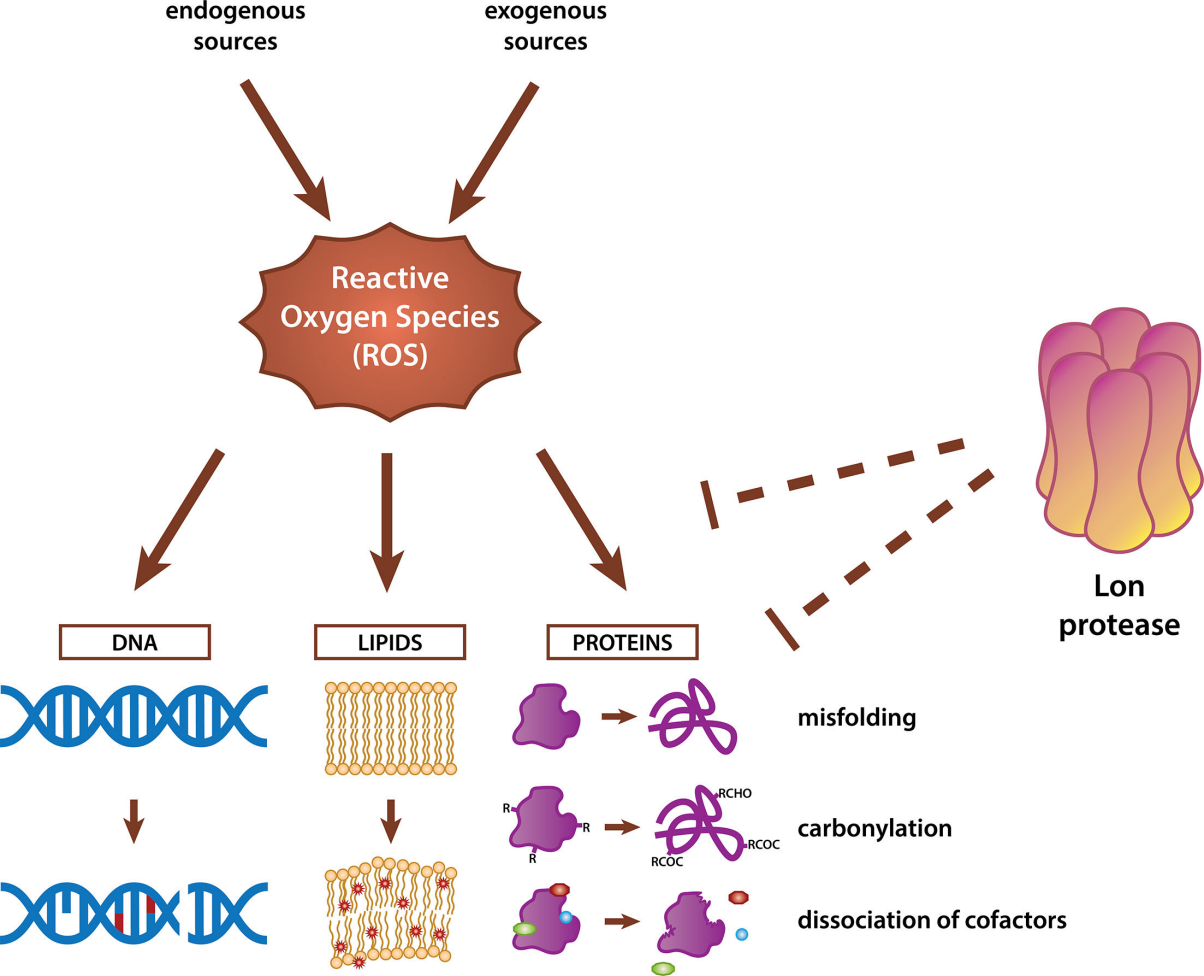
Overview of oxidative damage on macromolecules (DNA, lipids, and proteins) and the role of Lon protease in cellular defense against oxidative stress. Protective roles of Lon protease include regulation of oxidative stress responses (21, 22) and potential removal of oxidatively damaged proteins (8, 23–25).

Reactive oxygen species (ROS) are highly reactive molecules that contain oxygen, and they can be generated from both endogenous and exogenous sources. Endogenous sources include metabolic processes such as cellular respiration, organelle functions, and enzymatic reactions (12, 13). Among the endogenous sources of ROS, aerobic metabolism is considered the most inevitable source as partial reduction of oxygen during electron transport generates ROS in nearly all organisms (10, 14). Environmental pollutants, UV radiation, and toxins induce exogenous oxidative stress (15–17). For invading bacteria, the oxidative burst from macrophages constitutes an exogenous stress as well (18). To maintain a balance between ROS production and removal, the cells employ various antioxidant mechanisms, including enzymes and metabolites, to neutralize excess ROS (3, 4). In particular, hydrogen peroxide is scavenged by catalases and peroxidases, whereas superoxide is cleared by superoxide dismutase (3). Accumulation of ROS is known to damage various cellular components and macromolecules. For instance, ROS can harm DNA and lead to mutations, with the most common oxidative modifications being 7,8-dihydro-8-oxoguanine (8-oxoG) formation and single- or double-strand breaks (2, 19). ROS can also attack lipids, compromising their structural integrity and generating harmful products that can also induce oxidative modifications in proteins, exacerbating cellular oxidative damage (20) (Fig. 1).

Proteins are sensitive to oxidative damage through many routes including direct oxidation of amino acids, creation of disulfide bonds, and oxidation of metal cofactors and prosthetic groups (Fig. 1). ROS, including hydroxyl radicals (•OH) generated through the Fenton reaction, initiate oxidative modifications on specific amino acid residues (26). Among the sulfur-containing amino acids, cysteine is particularly vulnerable to oxidation by hydroxyl radicals and H_2_O_2_, serving as an indicator of redox status and regulating protein activity (10, 27). Aromatic amino acids undergo irreversible oxidative modifications when attacked by hydroxyl radicals, which can destabilize protein structure by altering hydrophobic interactions (26, 28–30). Another prominent oxidative modification on proteins is carbonylation, which involves the irreversible addition of reactive carbonyl groups (aldehydes and ketones) on certain amino acid side chains (e.g., lysine, arginine, proline, and threonine) via oxidation by ROS or reactive carbonyl species (RCS) (26, 30–32). The resulting carbonyl derivatives can participate in further chemical reactions, likely altering protein structure and increasing the risk of aggregation (31, 33, 34). Because the accumulation of damaged proteins could have disastrous effects on the cell, the clearance of these proteins is critical.

## THE LON PROTEASE

The Lon protease belongs to the AAA+ protein superfamily and is highly conserved across all kingdoms of life (11, 35). In *Escherichia coli*, Lon not only contributes to protein quality control but also regulates the cellular response to genotoxic stress and oxidative stress. It facilitates recovery from DNA damage by degrading the cell division inhibitor SulA and modulates oxidative stress by targeting the antioxidant stress response activator SoxS (21, 36). In *Caulobacter crescentus*, Lon is important for managing proteotoxic stress. It regulates cell division and deoxyribonucleotide production by degrading the replication initiator DnaA and the DNA adenine methyltransferase CcrM, respectively (37, 38). Lon also targets the general stress response sigma factor σ^T^, modulating the response to osmotic and oxidative stresses (39). Additionally, Lon is involved in regulating processes such as biofilm formation and motility, with its role varying across different species (40–43). In mitochondria, Lon contributes to mitochondrial DNA (mtDNA) compaction and transcription through its degradation of TFAM (44, 45). It also regulates the activation of mitochondrial unfolded protein response (mtUPR) by targeting ATFS-1 for degradation in healthy mitochondria (46).

Lon’s expression is induced by heat shock in bacteria, and it is considered a key component of the proteolytic machinery crucial for degrading abnormal proteins that accumulate under stress (47, 48). Lon degrades certain misfolded substrates in *E. coli*, including nonsense fragments of beta-galactosidase that feature a degradation motif containing hydrophobic amino acids typically buried within the protein core (49–51). Attachment of this motif to proteins normally not targeted by Lon promotes their degradation, suggesting that Lon can recognize proteins with exposed hydrophobic regions. Similarly, although mitochondrial Lon fails to recognize native, folded forms of ornithine transcarbamylase (OTC) and malate dehydrogenase (MDH), it successfully degrades chemically unfolded forms of both enzymes and a misfolded form of OTC (52). These findings reveal that Lon selectively targets proteins based on their folding state. How Lon recognizes these substrates is not known, but Lon’s recognition of exposed hydrophobic regions (49) and conformational changes that occur via oxygen-induced dissociation of cofactors (22, 53, 54) suggests a direct link between proteolysis and protein quality control, especially during stress.

## IMPORTANCE OF LON DURING OXIDATIVE STRESS

**Fig 2:**
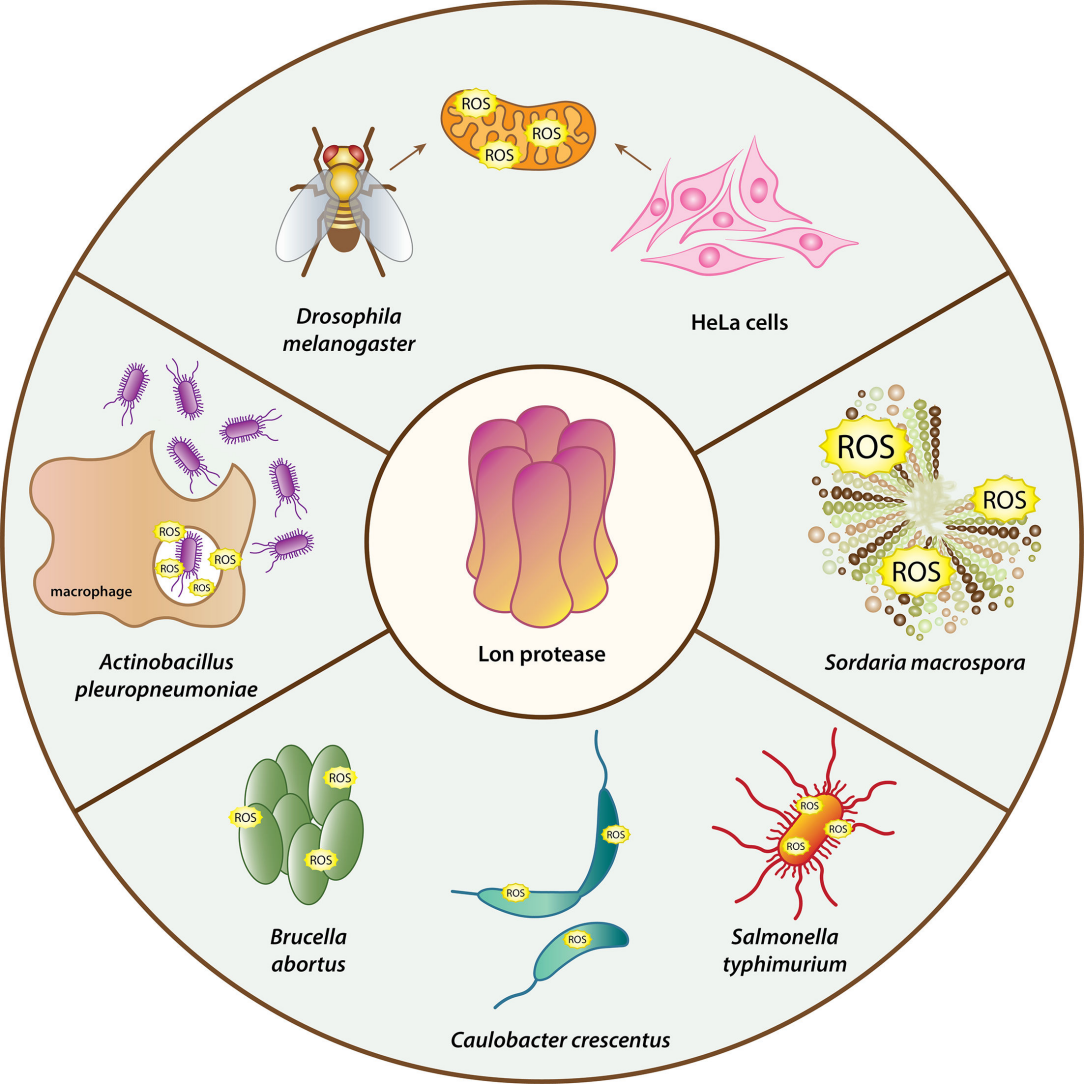
Organisms where Lon is involved in the defense against oxidative stress. Lon is important for maintaining mitochondrial ROS levels in *Drosophila melanogaster* and HeLa cells (62, 63), the survival of *Actinobacillus pleuropneumoniae* during oxidative stress likely arises from phagocytosis by macrophages (64), and the viability of *Sordaria macrospora*, *Brucella abortus*, *Caulobacter crescentus,* and *Salmonella typhimurium* during oxidative stress induced by H_2_O_2_ (65–67).

The Lon protease is crucial for the oxidative stress response in several systems. In the fungus *Sordaria macrospora* (*S. macrospora*), glyoxysomal Lon protease is associated with oxidative stress tolerance (55). Glyoxysomes are a specialized type of peroxisome found in plants and filamentous fungi, containing enzymes that are involved in fatty acid oxidation. *S. macrospora* encodes two Lon proteases, with LON2 predominantly located in glyoxysomes. Loss of this paralog results in several growth-related phenotypes, including heightened sensitivity to oxidative stress (Fig. 2). Furthermore, studies in several pathogenic and non-pathogenic bacteria indicate that Lon is important for defense against elevated ROS levels. *E. coli* resists oxidative stress through two key transcription factors, OxyR and SoxR (3). OxyR responds to even slight increases in intracellular H_2_O_2_ (56), whereas SoxR protects the cells against superoxide produced by redox cycling drugs (57). SoxR is activated upon oxidation of its iron-sulfur (Fe-S) cluster and triggers the production of SoxS, which regulates genes that confer resistance to superoxide (58–60). Once oxidative stress subsides, the system is deactivated through decreased *de novo* SoxS synthesis and degradation of existing SoxS by the Lon protease, emphasizing Lon’s role in the regulation of oxidative stress response in *E. coli* (21). Interestingly, a 21-amino acid segment at the N-terminus of SoxS serves as a recognition signal for Lon protease, driving its degradation (61). Attaching this segment to GFP renders it susceptible to Lon-dependent degradation, despite GFP itself not being a Lon substrate. These results indicate that Lon’s degradation of SoxS is not specific to oxidative stress, but rather, it is a result of the intrinsic instability of SoxS.

In *Salmonella typhimurium*, Lon regulates oxidative stress resistance through multiple mechanisms. *lon* mutants exhibit increased sensitivity to H₂O₂ damage (65) (Fig. 2) and upregulated expression of genes involved in iron import (68). As excess iron promotes hydroxyl radical formation and macromolecular damage (69–71), increased iron uptake in *lon* mutants may exacerbate oxidative damage. However, Lon’s degradation of catalase-peroxidase KatG indicates that Lon can also act to limit oxidative stress resistance (65). In *Actinobacillus pleuropneumoniae*, the absence of LonA sensitizes cells to oxidative stress (64), which is thought to arise when phagocytosed bacteria are exposed to a rapid release of ROS, causing substantial oxidative damage to bacterial cells (72) (Fig. 2). The sensitivity to H_2_O_2_ has also been observed in *lon* knockout strains of *Caulobacter crescentus* and *Brucella abortus*, both belonging to the α-proteobacteria division (66, 67) (Fig. 2). However, in these studies, H₂O₂ sensitivity is often accompanied by additional phenotypes, such as increased susceptibility to DNA damage or translation inhibition. In *Caulobacter crescentus*, the deletion of *lon* leads to slower growth, characterized by a prolonged lag phase and diminished cell mass accumulation during the stationary phase (42). *Brucella abortus lon* mutants have a growth defect at standard growth conditions (67). On the other hand, the *lonA* mutant of *Actinobacillus pleuropneumoniae* did not show any growth defect at 37°C but exhibited reduced growth at low (25°C) and high temperatures (42°C) (64). In *Salmonella*, deletion of *lon* does not change the growth characteristics (68). Loss of Lon is generally associated with reduced fitness and growth in many bacterial species, but the degree of impact varies dramatically (40–43, 64, 67, 73–75). Therefore, it remains unclear whether Lon directly contributes to oxidative stress tolerance or if the observed H₂O₂ sensitivity in these species results from a growth defect associated with the loss of *lon*.

## THE ROLE OF OXIDATIVE STRESS IN EXPRESSION OR ACTIVATION OF LON PROTEASE

Several studies suggest that the expression and activity of Lon protease are likely subject to regulation by oxidative stress itself. Exposure to H_2_O_2_ significantly increases Lon protein levels in the yeast *Saccharomyces cerevisiae* (*S. cerevisiae*) (76) and in human rhabdomyosarcoma cells (77). In contrast, although Lon is essential for adapting to H₂O₂ stress in *Drosophila melanogaster* (Fig. 2), its expression does not change after H₂O₂ treatment (62), indicating that the regulation of Lon’s expression by H_2_O_2_ may not be conserved among eukaryotes. In *Bacillus subtilis*, initial studies reported a significant increase in *lonA* mRNA levels during oxidative stress (78), but more recent findings suggest LonA protein levels remain unchanged during oxidative stress (22). This discrepancy implies that *B. subtilis* has sufficient Lon capacity to combat oxidative stress without needing to upregulate Lon. Surprisingly, although both *Brucella abortus* and *Caulobacter crescentus* strains lacking Lon protease are sensitive to H₂O₂, *lon* mRNA levels remain unchanged upon H₂O₂ treatment (67, 79). These findings suggest that although Lon is crucial for oxidative stress resistance, its expression is not regulated by increased H₂O₂ levels.

**Fig 3:**
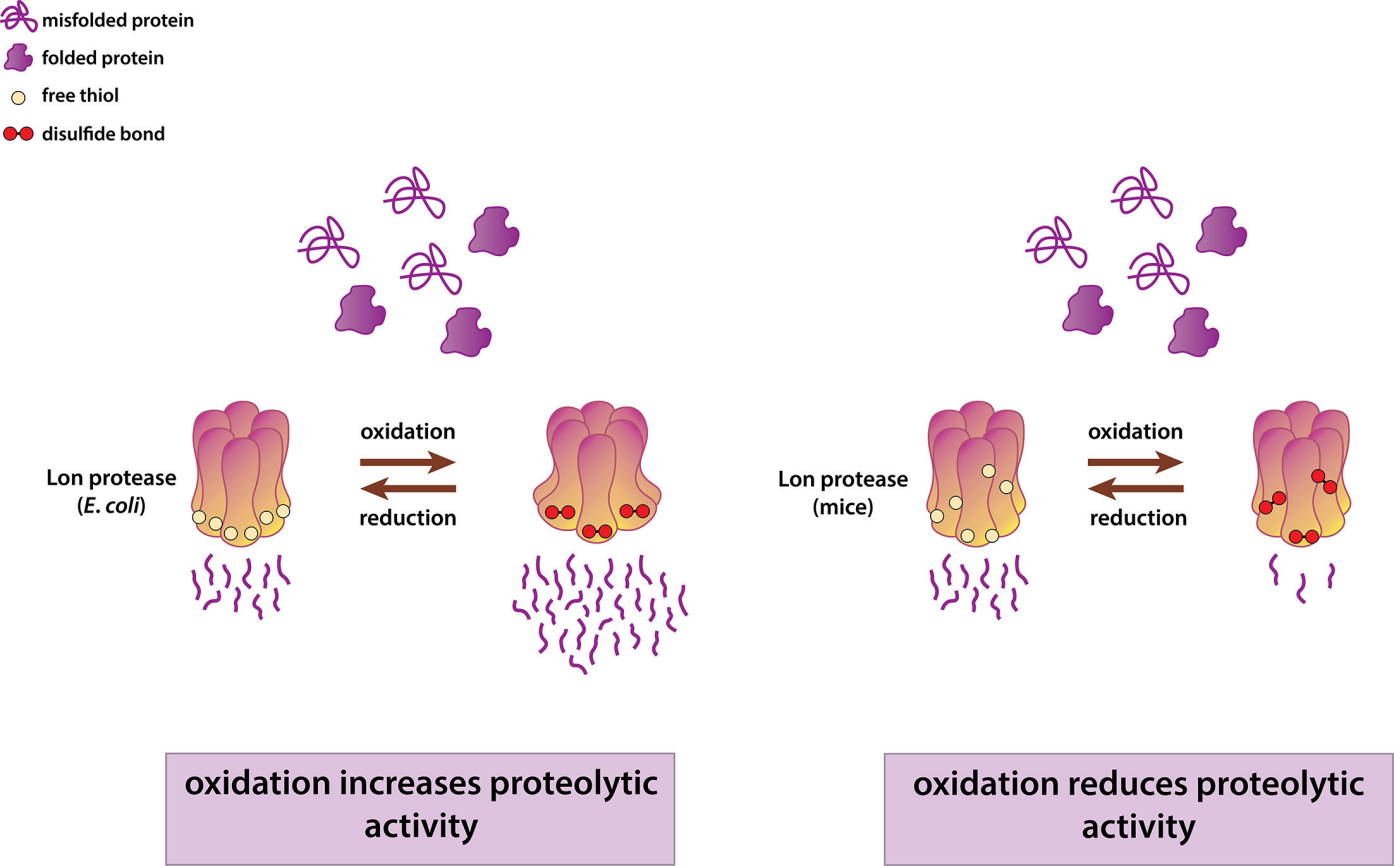
Cartoon depiction of Lon protease activation by oxidation in *E. coli* and by reduction in mice. Exposure of *E. coli* Lon to air or glutathione disulfide (GSSG) triggers the formation of an intrachain disulfide bond, which enlarges the exit pore and accelerates proteolysis (80). In contrast, Lon protease activity in aging mouse models decreases due to oxidation but can be restored through reduction (81).

More recently, a surprisingly direct molecular mechanism of Lon regulation during oxidative stress was discovered in *E. coli* (80). Specific cysteine residues are conserved among the Lon orthologs from the *Enterobacteriaceae* family of gram-negative bacteria. In *E. coli*, a pair of these conserved residues forms a disulfide bond under aerobic conditions and alters the structure of the proteolytic domain of Lon. The introduction of dithiothreitol (DTT) reduces this disulfide bond, whereas glutathione disulfide (GSSG) promotes its formation. This structural change widens peripheral pores in the peptidase domain, resulting in a more active Lon peptidase chamber. Without this disulfide bond, these pores are narrowed and restrict peptide exit, reducing Lon’s activity. This redox-dependent regulation of Lon activity is crucial for the survival of enteric bacteria inside and outside the host. The reductive, anaerobic environment of the host gut keeps Lon in a reduced state, whereas exposure to oxygen outside the host shifts it to an oxidized form (Fig. 3). Since oxygen generates reactive oxygen species that damage biomolecules, the increased Lon activity under oxidative and aerobic conditions aligns with an often proposed role in degrading misfolded and damaged proteins that accumulate in these conditions.

Interestingly, the opposite effect has been seen in mice treated with 1-methyl-4-phenyl-1,2,3,6-tetrahydropyridine (MPTP), an oxidant which induces Parkinson-like symptoms (82). Although MPTP treatment increased Lon protein levels, its proteolytic activity remained unchanged (81), indicating reduced Lon activity. The addition of the reducing agent dithiothreitol (DTT) restored Lon’s activity, suggesting that MPTP inactivates Lon by oxidizing specific thiol groups, and enzyme reactivation occurs upon reduction (Fig. 3). In addition, treatment of human mitochondrial Lon protease (LONP1) with H_2_O_2_ diminished both ATP hydrolysis and proteolytic activity *in vitro* (83). However, this treatment also caused irreversible oxidative damage to Lon, suggesting that reduced activity results from oxidation of amino acid residues crucial for its function. Therefore, it is possible that the H_2_O_2_ concentration used in the study was too high to reveal whether Lon activity is normally regulated by oxidation. These studies illustrate that oxidative stress might regulate Lon protease activity through distinct mechanisms across organisms. In *E. coli*, oxidation activates Lon by widening the exit pore through the formation of disulfide bonds, whereas in mice, disulfide bond formation can inactivate Lon. These contrasting effects suggest that redox-dependent regulation of Lon’s activity does not appear to be conserved.

## DEGRADATION OF PROTEINS BY LON DURING OXIDATIVE STRESS

**Fig 4:**
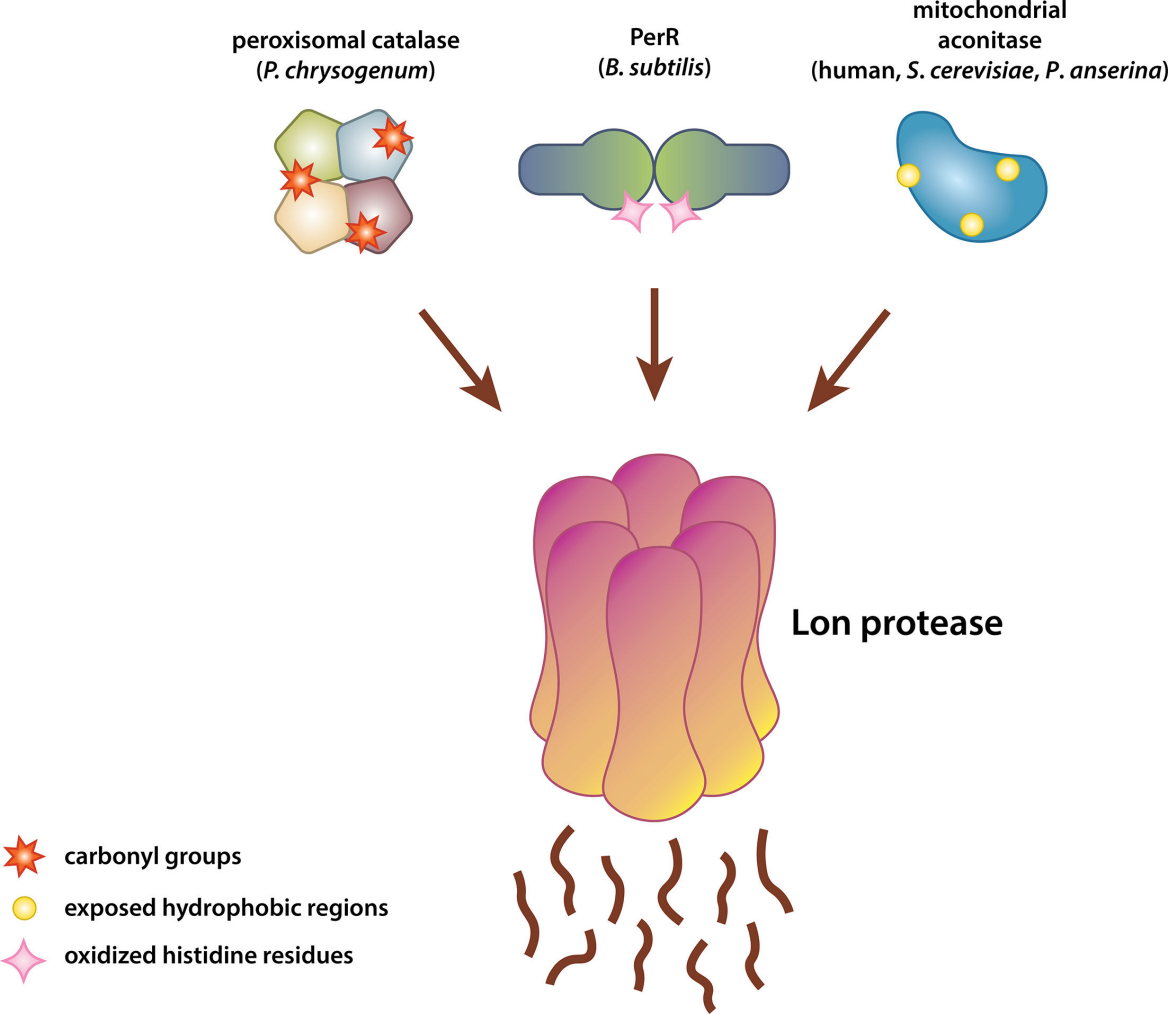
Known oxidized substrates of Lon protease in several different species. Lon degrades oxidatively damaged forms of peroxisomal catalase of *Penicillium chrysogenum* (88), PerR of *B. subtilis* (22), and mitochondrial aconitase of humans, yeast (*S. cerevisiae*), and fungus (*Podospora anserina*) (23, 76, 89).

The need to remove damaged proteins following oxidative stress is evident, but the role of Lon in this process is unclear. Early evidence of a proteolytic system directly involved in degrading oxidatively damaged proteins came from mitochondria, where the addition of matrix extracts caused breakdown of oxidatively modified proteins by a pH-sensitive, metal-dependent serine protease (84). A similar study in *E. coli* identified a proteolytic pathway specifically targeting oxidatively denatured forms of catalase, superoxide dismutase, albumin, and hemoglobin, generated by exposure to reactive oxygen species (ROS) (85). In these cases, proteolysis was not ATP-dependent, suggesting that a serine protease other than Lon was responsible for degrading oxidatively denatured proteins. Similarly, early studies in *E. coli* showed that oxidatively damaged inactive glutamine synthetase was preferentially degraded by Protease So, rather than Protease La (Lon) (86). Surprisingly, a membrane-bound Lon protease with ATP-independent activity for unfolded substrates and ATP-dependent activity for folded substrates has been discovered in the archaeon *Thermococcus kodakaraensis* (87). This proteolytic activity is most effective at basic pH and requires metal ions, displaying similarities to the proteolytic systems responsible for degrading oxidized proteins in mitochondria and in *E. coli*. This suggests that the proteolysis of oxidized proteins in these organisms may still rely on Lon protease. In support of a role for Lon in directly destroying oxidatively damaged proteins, it was shown that the mammalian mitochondrial Lon (LONP1) degrades mildly oxidized mitochondrial aconitase in an ATP-dependent manner (23) (Fig. 4). Reduction of *Lonp1* expression using antisense oligonucleotides diminished the mitochondrial matrix’s capability to degrade both oxidized and native aconitase. Similarly, *in vitro* experiments showed that ATP addition significantly enhances proteolytic activity, and oxidized aconitase is more readily recognized by the Lon protease. Degradation of damaged aconitase was also seen in yeast mitochondria, with ATP-dependent degradation significantly increasing upon treatment with menadione, which induces superoxide stress, but not with H_2_O_2_ (76).

There are examples of Lon degrading more specific substrates during oxidizing conditions. For example, in *B. subtilis*, the Lon ortholog LonA targets the oxidized form of PerR for degradation (22) (Fig. 4). PerR is an H_2_O_2_-responsive repressor of antioxidant genes (90). Upon an increase in cellular H_2_O_2_ levels, specific histidine residues undergo oxidation, leading to a loss of metal binding (56). Release of the metal-free PerR from DNA and its subsequent degradation by LonA thus promotes the transcription of genes involved in oxidative stress response. Interestingly, both oxidized PerR and nonoxidized metal-free PerR are degraded by LonA and exhibit similar structures. This suggests that recognition by LonA does not depend on the oxidation state of the protein but rather on the absence of bound metals. Evidence indicates that oxidation increases the hydrophobicity of aconitase (23); however, one study proposed that the degradation of aconitase by LONP1 in mammalian mitochondria might also occur due to oxidation of its Fe-S cluster (91). These studies point to oxidation of Fe-S clusters and loss of cofactor binding as a degradation signal for Lon-mediated proteolysis, but it remains uncertain whether these signals are sufficient on their own to dictate degradation.

## ROLE OF LON IN REGULATING CARBONYLATED PROTEIN LEVELS

A well-known modification of proteins by ROS is the carbonylation of residues, often leading to misfolding or aggregation. In *E. coli*, accumulation of hydroxyl radicals can lead to increased carbonylation of proteins during the stationary phase (92, 93). Deletion of *lon* further increases the accumulation of carbonylated proteins, whereas overexpressing DnaK/DnaJ reduces their levels (24). These findings suggest a model in which the DnaK/DnaJ chaperone system helps clear carbonylated proteins by maintaining them in a soluble state for subsequent degradation by Lon protease. However, if the accumulation of carbonylated proteins exceeds a certain threshold, it can overwhelm the protein quality control system, leading to the formation of aggregates that resist degradation.

The loss of Lon (PIM1) leads to a significant increase in carbonylated protein levels in *S. cerevisiae* and accumulation of several oxidized proteins, including subunits of ATP synthase (8). Likewise, genetic silencing of *lonp1* in rhabdomyosarcoma (RD) and HeLa cells results in elevated levels of carbonylated proteins (63, 77). Similarly, loss of Lon protease activity in rat brains was associated with elevated levels of carbonylated proteins (94), and senescent WI-38 cells show reduced Lon levels and increased carbonylated proteins compared with earlier passage cells (25).

In contrast, the absence of mitochondrial Lon (Lon1) in *Arabidopsis thaliana* does not change the global carbonylation profile in mitochondria (95). Although there is no evidence of increased oxidative damage in the absence of Lon1, deletion of *LON2*, the peroxisomal Lon protease, results in the upregulation of antioxidant genes such as catalase (96). Whether Lon2 is directly involved in oxidative stress resistance in *Arabidopsis* is not known; however, Lon is shown to be important for maintaining peroxisomal protein quality during oxidative stress in other organisms (97). For example, in *Penicillium chrysogenum* (*P. chrysogenum*), a filamentous fungus, the peroxisomal Lon protease, referred to as Pln, is responsible for the degradation of oxidatively damaged heme-bound catalase-peroxidase to preserve peroxisome function (88) (Fig. 4).

Finally, in *P. anserina*, constitutive expression of mitochondrial Lon (PaLON) leads to reduced accumulation of carbonylated and carboxymethylated proteins (89). This filamentous fungus is a classic model organism for studying aging, as its senescence is closely linked to mitochondrial health (98, 99). Compared with other organisms, aging in *P. anserina* results in elevated carbonylation of specific proteins, such as aconitase, rather than an overall increase in protein carbonylation. Levels of carbonylated aconitase decrease upon overexpression of *PaLON*, suggesting that PaLON degrades oxidatively damaged aconitase, similar to the mammalian Lon (Fig. 3). Interestingly, overexpression of active PaLON extends the lifespan of *P. anserina* compared to the wild-type strain, likely by reducing the accumulation of ROS and oxidatively damaged proteins.

## HEME AND FE-S REGULATION BY LON DURING OXIDATIVE STRESS

Electron transport chain complexes and some ROS-scavenging enzymes, such as catalases, require heme or Fe-S clusters as cofactors. Although the reduction in cellular free iron pools is necessary during oxidative stress to prevent ROS generation through Fenton chemistry, it poses challenges for heme and Fe-S cluster synthesis (100). Consequently, the tightly regulated production of heme is a common feature in both eukaryotic and prokaryotic organisms. Numerous studies indicate the involvement of the Lon protease in regulating heme biosynthesis at various stages.

In mammals, the accumulation of heme initiates a regulatory mechanism leading to the suppression of mitochondrial 5-aminolevulinic acid (ALA) synthase (ALAS-1), the first enzyme of the heme biosynthetic pathway (101). Excessive heme not only attenuates gene transcription (102, 103) and mitochondrial import of the pre-protein (104) but also amplifies the rate of ALAS-1 degradation. Inhibition of LONP1 or silencing *lonp1* prevents heme-dependent degradation of ALAS-1, indicating Lon’s involvement in this process (105). A recent study demonstrated that mitochondrial ClpX (mtClpX) also contributes to ALAS-1 degradation (106). Elevated heme levels promote complex formation between mtClpX and ALAS-1, and mtClpX deficiency results in ALAS-1 accumulation. Elevated heme levels promote a complex formation between mtClpX and ALAS-1, and mtClpX deficiency leads to ALAS-1 accumulation. Notably, hemin addition increases ALAS-1 carbonylation, indicating that heme binding enhances protein oxidation. Although the levels of ClpP and ClpX in ALAS-1 immunoprecipitates remain unchanged with hemin treatment, the amount of LONP1 increased. These results suggest that mtClpX primarily targets heme-bound ALAS-1, whereas LONP1 may degrade its oxidatively damaged form, although hemin addition could be affecting LONP1 binding in some other manner as well. In *Salmonella typhimurium*, both Lon and ClpAP proteases target HemA, the enzyme catalyzing the first step of bacterial heme biosynthesis (107, 108). Similar to ALAS-1 degradation by LONP1, HemA degradation occurs when cellular heme levels are sufficient. It remains unclear whether ClpAP, similar to mtClpXP, degrades HemA early on, whereas Lon degrades its oxidatively damaged form. Nonetheless, the observed cooperation in regulating heme biosynthesis in both mammalian mitochondria and *Salmonella* emphasizes the pivotal role of proteases in maintaining this balance.

Lon’s recognition of heme-bound enzymes is also seen in liver mitochondria, where LONP1 degrades cystathionine β-synthase (CBS) upon oxygenation of its bound heme (109). During ischemia or hypoxia, deoxygenation of heme prevents recognition by LONP1 and promotes CBS accumulation. This accumulation, in turn, depletes H_2_S, an important scavenger of ROS, suggesting that Lon has a protective role against oxidative stress. Heme oxygenase-1 (HO-1) also undergoes Lon-mediated proteolysis in a similar manner. Although this degradation is not dependent on the cell’s redox status, Lon’s role in controlling the levels of both heme-producing and heme-degrading enzymes in various conditions suggests that Lon protease is essential for maintaining appropriate heme levels.

In *Caulobacter crescentus*, Lon-mediated proteolysis of FixT regulates heme production. FixT, a negative regulator of the FixL-J two-component regulatory system, limits heme biosynthesis through inhibition of FixL (53, 110). Recognition of FixT by Lon depends on the status of its Fe-S cluster. Upon oxygenation, the Fe-S cluster dissociates, promoting FixT degradation. This degradation then allows FixL to become active and activate transcription of genes involved in heme biosynthesis. Although Lon’s degradation of FixT indirectly influences cellular heme levels, a recent study indicates that the H_2_O_2_ sensitivity in ∆*lon* cells is not restored upon loss of *fixT*, indicating that limited heme production is not the underlying cause (66). The precise mechanism by which Lon deficiency leads to growth defects during oxidative damage in *Caulobacter* remains unknown.

Lon protease not only regulates heme biosynthesis but also plays a role in iron import in *Salmonella typhimurium*. Ferrous iron (Fe(II)) import in Salmonella is mediated by the Feo system, which includes FeoA, FeoB, and FeoC (111). FeoB, an inner membrane transporter, facilitates Fe(II) uptake through GTP hydrolysis. Under low-oxygen, low-iron conditions, FeoC binds FeoB to protect it from degradation by FtsH protease (112). Interestingly, FeoC itself is regulated by proteolysis. When iron and oxygen levels are high, FeoC is degraded by Lon, preventing FeoB accumulation (54). Lon recognizes FeoC in Salmonella similarly to its recognition of FixT in Caulobacter, involving oxygen-induced dissociation of Fe-S clusters. However, mutating FeoC’s Fe-S binding cysteines to alanine increased its stability under high-oxygen conditions, whereas a similar mutation accelerated FixT degradation by Lon. This suggests that although Fe-S clusters stabilize FixT, they act as degradation signals for FeoC when oxidized. Despite these differences, Fe-S clusters clearly play a crucial role in regulating oxygen-dependent pathways through Lon-mediated proteolysis. Notably, *Lon* deletion sensitizes *Salmonella* to H₂O₂, likely due to the upregulation of iron uptake genes (68). In high-oxygen conditions, the absence of Lon may prevent FeoC degradation, leading to persistent Feo system activation. This could result in excessive iron uptake and increased ROS generation via the Fenton reaction, thereby heightening susceptibility to oxidative stress.

## DISCUSSION

Lon protease stands as a critical regulator of cellular homeostasis and adaptive responses, particularly in the face of oxidative stress, which poses a significant threat to cellular integrity across diverse organisms. In both eukaryotes and prokaryotes, damaged proteins accumulate under oxidative stress, disrupting cellular functions. Oxidative damage to proteins causes direct oxidation or carbonylation of amino acid residues, resulting in disruption of protein structure and potential recognition by Lon. Based on Lon’s degradation of oxidized aconitase (23) and the accumulation of carbonylated proteins in its absence (8, 63), the prevailing model proposes that Lon selectively targets oxidatively damaged proteins. However, these results do not definitely show that oxidation of proteins is sufficient to trigger their degradation by Lon. Indeed, there are a few examples of oxidized proteins that Lon fails to recognize (85) and instances where the loss of Lon does not result in the accumulation of carbonylated proteins (95). In addition, the direct evidence that Lon degrades carbonylated proteins is insufficient. The absence of Lon correlates with the accumulation of carbonylated proteins in various species (24, 25, 63), and it is tempting to link this increase in steady-state levels of carbonylated proteins to a loss of Lon-dependent proteolysis. However, the absence of Lon protease could also impact other protein quality control systems, antioxidant pathways, or even ROS accumulation, as observed in both *Salmonella* (68) and in HeLa cells (63), which can lead to heightened oxidative damage to proteins and subsequent carbonylation of amino acid residues. Therefore, further research is necessary to determine whether Lon specifically targets carbonylated proteins.

Assuming that carbonylated proteins are degraded by Lon, it still remains unclear whether Lon has a distinct mechanism to target them or if their recognition is a result of protein misfolding due to carbonylation. Additionally, it is possible that Lon identifies certain proteins during oxidative stress via different mechanisms, such as the dissociation of cofactors or bound metals that occurs as a result of oxidation (22). The different modifications on proteins resulting from oxidative damage can each serve as a degradation signal for Lon-mediated proteolysis. Therefore, even if Lon plays a role in the degradation of oxidatively damaged proteins, determining whether a universal mechanism exists for the recognition of these proteins remains a challenge.

We therefore argue that Lon’s regulation of oxidative stress response in organisms is not through a singular mechanism. Although Lon certainly degrades some oxidized proteins, it remains undetermined whether it also targets carbonylated proteins or how it recognizes them. Aside from its role in protein quality control during stress, Lon is also crucial for cellular survival during oxidative stress. It regulates antioxidant pathways, such as through degradation of SoxS in *E. coli* (21) or PerR in *B. subtilis* (22), as well as balancing iron homeostasis in different organisms. It modulates the stability of key enzymes involved in heme biosynthesis and iron import through recognition of heme-bound and Fe-S cluster-containing proteins in an oxygen-dependent manner (53, 54, 109). As the balance of intracellular heme and iron levels is crucial during oxidative stress, these combined functions of Lon position it as a key component of cellular resilience against oxidative stress. Understanding the mechanisms by which Lon recognizes and degrades its oxidized substrates, as well as how its activity adapts to oxidative conditions, would reveal new insights into our understanding of its function. Ultimately, further research into Lon’s diverse roles will enhance our comprehension of how cells utilize Lon protease for their survival during stress, contributing to potential advancements in treatments for diseases linked to oxidative damage.
